# Acknowledgment to the Reviewers of *Dermatopathology* in 2022

**DOI:** 10.3390/dermatopathology10010007

**Published:** 2023-01-19

**Authors:** Dermatopathology Editorial Office

**Affiliations:** MDPI AG, St. Alban-Anlage 66, 4052 Basel, Switzerland

High-quality academic publishing is built on rigorous peer review. *Dermatopathology* was able to uphold its high standards for published papers due to the outstanding efforts of our reviewers. Thanks to the efforts of our reviewers in 2022, the median time to first decision was 40 days and the median time to publication was 63 days. Regardless of whether the articles they examined were ultimately published, the editors would like to express their appreciation and thank the following reviewers for the time and dedication that they have shown *Dermatopathology*:
Adam I. RubinMa Teresa Fernández FiguerasAndrzej SlominskiMaged DaruishAngelo RuggieroMar Llamas-VelascoAshley N. ElsensohnMarie-Charlotte BrüggenCandice BremMaxwell A. FungCarlo CotaMeenakshi BatraniDeepika YadavMichelangelo La PlacaDennis NiebelMichelle Weiting LiangDieter MetzeMuhammad N. MahmoodEiji KiyoharaNadia ShiraziFrancisco BravoPanitta SithinamsuwanG. NandakumarPaola SavoiaGianluca NazzaroPhilip E. LeboitGiorgio FilosaPhyu P. AungGiovanni PaolinoPierre A. De ViraghGiusto TrevisanRahul MannanHans-Joachim SchulzeRajalakshmi TirumalaeHeinz KutznerRenee HowardHelmut BeltraminelliRino CerioHui Yi ChiaSadia AfrinJean KanitakisSanaullah SajibJeffrey P. NorthShang-Ian TeeJivko A. KamarashevShin-Ichi OsadaJoel Hua Liang LimSuat Hoon TanJosé Carlos CardosoTakemichi FukasawaJose Manuel MascaroTatsiana PukhalskayaJosef SmolleTeresa GriecoKara E. YoungTeresa LettiniKlaus EisendleThuylinh PhungLeonardo RestaUwe WolinaLeonie BeyerVenkatesh RameshLionel FontaoWayne GraysonLiuyan JiangWei-Ti Chen


